# Ethical competence: the cornerstone of care in the cardiac operating room, a phenomenological study

**DOI:** 10.1186/s12910-025-01295-1

**Published:** 2025-10-08

**Authors:** Seyed Kianoosh Hosseini, Fateme Mohammadi, Seyed Reza Borzou, Mostafa Bijani, Seyedeh Zahra Masoumi

**Affiliations:** 1 Department of Cardiology, School of Medicine, Farshchian Cardiovascular Subspecialty Medical Center, Hamadan University of Medical Sciences, Hamadan, Iran; 2 Department of Pediatric Nursing, School of Nursing and Midwifery,Mother and Child Care Research Center Institute of Health Sciences and Technology, Hamadan University of Medical Sciences, Hamadan, Iran; 3 Department of Medical-Surgical Nursing, School of Nursing and Midwifery, Chronic Diseases (Homecare) Research Center, Department of Nursing, Hamadan University of Medical Sciences, Hamadan, Iran; 4 Department of Medical-Surgical Nursing, Fasa University of Medical Sciences, Fasa, Iran; 5 Department of Midwifery, School of Nursing and Midwifery,Mother and Child Care Research Center Institute of Health Sciences and Technology, Hamadan University of Medical Sciences, Hamadan, Iran

**Keywords:** Moral competency, Operating room, Cardiovascular, Qualitative study

## Abstract

**Background:**

Ethical competence leads to the provision of principled and ethical care, which subsequently improves health and increases patient satisfaction. Among these, the cardiac operating room is one of the most challenging and stressful therapeutic environments, in which the ethical competencies of healthcare professionals can significantly impact the performance and quality of care. Therefore, it is critical to describe healthcare professionals’ experiences in the operating room regarding ethical competency. The present study aimed to investigate the experiences and perceptions of ethical competency among healthcare professionals in a cardiac operating room.

**Methods:**

This qualitative research project utilized a phenomenological design and included 23 participants selected through purposeful sampling. Data were collected from July 2023 to March 2024. Sampling continued until data saturation was achieved. Data were collected through semi-structured interviews. The collected data were analyzed using the Colaizzi approach.

**Results:**

The mean age of the participants was 36.64 ± 3.53 years. Three final themes with ten theme clusters “respect to the dignity of the patient (respecting the patient’s identity, respecting physical and sexual privacy, protecting information privacy), promoting the patient’s mental health (paying attention to the patient’s independence, providing comprehensive information, compassion) and expansion of moral obligations (promoting moral courage, avoiding discrimination, expansion ethical and principled care, promotion of ethical knowledge) were extracted from the data.

**Conclusion:**

According to this research, respecting the dignity of patients, paying attention to their identity and privacy, and promoting mental health and professional support are the most important indicators of ethical competence, which are associated with developing moral courage and fair and thoughtful decision-making in healthcare professionals. Based on this, officials and policymakers can take fundamental steps towards achieving and promoting the ethical competence of healthcare providers by creating fair policies that preserve human dignity and adhere to the principles of professional ethics.

**Clinical trial number:**

Not applicable.

## Introduction

Ethical competence is crucial in healthcare systems [1, 2] because the primary mission of healthcare professionals is to deliver high-quality and ethical care in the areas of prevention, treatment, and rehabilitation that promote the well-being of individuals in society [1, 3, 4]. Ethical competence for healthcare professionals is defined as having ethical knowledge, respecting individual and cultural differences, and having the skills and abilities to deal with ethical problems [1]. Ethical competence is an abstract and multidimensional concept influenced by various cognitive, emotional, behavioral, cultural, economic, personality, and therapeutic factors [5–7].

Therefore, it is essential that healthcare professionals continually develop the ethical competence to provide principled care [1, 3, 4]. This is while the operating room is one of the most important clinical environments where the ethical competence of healthcare providers is crucial [8].

Patients in the operating room experience physical and psychological tension due to entering alone and without accompaniment in an unfamiliar environment filled with numerous medical devices [9, 10]; hence, they require psychological support and respectful and fair behavior from health care professionals [10]. Therefore, healthcare professionals in the operating room must have ethical competence to provide ethical and principled services [9]. Meanwhile, the cardiac operating room is one of the most important operating rooms, which faces a high patient load [11].

Blomberg et al. (2018) stated in their study that healthcare professionals preparing patients for surgery in the operating room must possess clinical knowledge, skills, commitment, and moral competency [12]. Similarly, Bilic et al. (2017) emphasized the importance and necessity of the ethical responsibility of healthcare professionals in the operating room. Accordingly, health policymakers and managers should plan appropriate programs to develop personnel’s ethical competency [13]. Although these studies explain and describe the importance of ethical competence of healthcare providers in the operating room, there is no study available to researchers that has examined the ethical competence of healthcare providers in the cardiac operating room. On the other hand, ethical competence can be influenced by clinical environments and the cultural context prevailing in the society.

Accordingly, the current study aimed to investigate healthcare professionals’ experiences and perceptions of ethical competency in the cardiac operating room.

## Materials and methods

### Study design and setting

This study was a qualitative study with a descriptive phenomenological approach that was conducted from July 2023 to March 2024 at the University of Medical Sciences in Western Iran. A qualitative study with a descriptive phenomenological approach was used to answer the research question, “What are the lived experiences of healthcare professionals regarding ethical competency in the cardiac operating room?”. Descriptive phenomenology is a qualitative research method that focuses on studying an individual’s lived experiences within the world and expresses the individual’s understanding, feelings, perspectives, and perceptions of a specific phenomenon or concept [14]. This study follows the Standards for Reporting Qualitative Research (SRQR) guidelines for qualitative research.

### Participants

In this study, thirty-four healthcare professionals were available. After receiving ethics approval, they were invited to participate in the study. Twenty-three healthcare professionals, who met the study inclusion criteria, were selected through purposive sampling and invited to this study. Purposeful sampling is commonly used in qualitative research to identify participants who can provide rich information on the phenomenon under study. Studying information-rich cases results in an in-depth understanding of the research topic, rather than empirical generalizations [15]. The inclusion criteria for the study included willingness to participate, having a minimum of 12 months of experience in cardiac operating rooms, familiarity with medical terminology, and the ability to provide detailed information.The exclusion criterion was unwillingness to continue the interview.

### Data collection

This study used interviews and field notes to collect data. Twenty-three semi-structured interviews were conducted face-to-face in quiet environments with the cooperation and willingness of participants. Each interview began with a few general questions, including “Can you describe a day of your work in the cardiac operating room?“,” What does ethical competence mean to you?”, “What does ethical competence mean to you?”. Subsequently, based on the respondents’ answers, follow-up questions would be asked to increase the clarity of the information—the questions included, “Can you explain further?“, “What do you mean by that?“, and “Can you give an example?“. The interviews were oriented around the main objective of the study. The interview questions were reviewed by 10 faculty members familiar with qualitative research, and the validity of the interview questions was confirmed. Each interview lasted between 50 and 70 min. The interviews were recorded, and immediately after each interview, the interviews were listened to by the corresponding author several times to develop a general understanding and deep insights, and then were transcribed on paper, along with the field notes. After each interview, data analysis was carried out, and the next interview was scheduled. Interviews continued until no other new sub-themes were created, and data reached Saturation.

### Data analysis

The author of the correspondence conducted and analyzed all interviews. Each interview was organized using MAXQDA software. The collected data were analyzed according to Colaizzi’s method, which consists of seven steps: (1) reading and re-reading each transcribed interview, (2) extracting important terms and phrases from the transcripts, (3) assigning meaning to the extracted units, (4) organizing and categorizing similar units, (5) presenting comprehensive descriptions of the extracted theme clusters, (6) creating final themes for the subject under study according to the extracted theme clusters, and (7) confirming the basic paradigm by having the participants verify the theme clusters and final themes [16]. The interviews were recorded and carefully reviewed by the main author to gain thorough understanding and insights. The interviews were then transcribed using field notes. Key terms and phrases were identified, and meaningful units were established. Theme clusters and final themes began to surface throughout the interviews, and member and peer checks were conducted to validate the data. The interviews proceeded until no new sub-themes emerged and the data reached saturation. Following each interview, a memo was created based on thought-provoking questions. Table 1 illustrates an example of the coding and progression of theme clusters and final themes.

**Table 1:** An example of coding and development of themes cluster and final themes

Meaning units	Coding	Themes cluster	final Theme
*Error is an inevitable part of medical treatment. Courage in reporting mistakes and shortcomings is a sign of commitment to ethical obligations in medical professions. I made a mistake regarding a patient’s illness. Although it was hard*,* I reported my mistake to my superior with all the details. I believe having courage is the most important indicator of ethical commitment and competence” (Participant 6*,* 39 years old).*	The Inevitability of Error “Courage” an Indicator of Adherence to Professional Ethics	Promoting moral courage	***Expansion*** **of moral** ***obligations***
*In government-operated healthcare facilities with insurance coverage*,* all patients should be afforded equal rights to access services. The patient’s economic*,* social*,* and political status should not influence the treatment process.” (Participant 3*,* 52 years old)*	Same rights to receive services Countering discrimination Countering discrimination	Avoiding discrimination	

### Rigor

Accordingly, to increase the credibility and accuracy of the data, the researchers used a combination of sources, semi-structured interviews, prolonged engagement with the data, four participants for member checks, and five expert researchers in qualitative studies for peer checks. Finally, Confirmability was acquired through the exact recording of participant narratives and detailed reporting of the study, to provide the possibility of follow-up for other researchers.

### Ethics approval and consent to participate

The Hamadan University of Medical Science, the Chronic Diseases (Home Care) Research Center's institutional review board provided ethics approval (IR.UMSHA.REC. 1402.261). The research code for the current study is 140207256120. All methods were performed in accordance with the relevant guidelines and regulations, and all the research methods met the ethical guidelines described in the Declaration of Helsinki. Also, at the beginning of each interview, the researcher introduced herself, explained the study's goals, and provided assurance that all information would remain confidential and that they could withdraw from the study at any time. The researchers allowed participants to inform the researcher about their withdrawal from the study at any stage. They assured them that their lack of participation or withdrawal would not have any consequences for them. Finally, the written informed consent was obtained from study participants. 

## Results

### Demographic information

The study included 23 participants (11 females and 12 males. The average age of the participants was 36.64 ± 3.53 years. Demographic information is shown in Table 2. Three final themes, including “respect to the dignity of the patient, “” promoting the patient’s mental health, “ and “expansion of moral obligations,” with ten theme clusters were extracted from the data. (Table 3.)

**Table 2:** Individual social characteristics of the participants

Participants	Sex	Age (year)	Marital status	Educational level	Work experience (years)
P1	Man	31	Married	Bachelor of nursing	7
P2	female	42	Married	Master of nursing	17
P3	Man	52	Married	Cardiology specialist	21
P4	female	27	Married	Bachelor’s degree in operating room	4
P5	Man	32	Married	Bachelor’s degree in operating room	8
P6	female	39	Single	Cardiology specialist	12
P7	female	34	Married	Bachelor’s degree in operating room	10
P8	Man	38	Married	Master of nursing	9
P9	Man	32	Married	Bachelor of nursing	10
P10	Man	44	Married	Bachelor of nursing	16
P11	female	32	Married	Bachelor’s degree in operating room	5
P12	Man	47	Married	Bachelor of nursing	15
P13	Man	27	Single	Bachelor of nursing	3
P14	female	24	Married	Bachelor of nursing	2
P15	female	28	Single	Bachelor of nursing	3
P16	Man	46	Single	Cardiology specialist	12
P17	female	40	Married	Cardiology specialist	17
P18	Man	50	Married	Bachelor of nursing	22
P19	female	25	Married	Master of nursing	3
P20	Man	39	Single	Master of nursing	11
P21	female	36	Married	Bachelor's degree in operating room	6
P22	Man	27	Single	Bachelor of nursing	2
P23	female	29	Single	Master of degree in operating room	5

**Table 3:** Theme clusters and themes in this study

***Respecting the patient’s dignity***	Respecting the patient’s identity
	Respecting physical and sexual privacy
	Protecting information privacy
***Promoting the patient’s mental health***	Paying attention to the patient’s independence
	Providing comprehensive information
	Compassionate
***Expansion*** **of moral** ***obligations***	Promoting moral courage
	Avoiding discrimination
	Expanding ethical and principled care
	Promotion of ethical knowledge

### Theme 1: Respecting the patient’s dignity

The participants in this study emphasized that respecting patients is one of the most important principles of professional ethics and a crucial factor in achieving ethical competence. Therefore, it is essential to pay attention to the religious and ethnic identities of patients and appropriately address them with local dialects and accents. Additionally, after obtaining permission, patients’ physical and sexual privacy should be respected, and their information should be kept confidential. Respecting the patient’s dignity includes three theme clusters: respecting the patient’s identity, respecting their physical and sexual privacy, and maintaining their confidentiality.

### Respecting the patient’s identity

The study participants emphasized that in the cardiac operating room, healthcare professionals’ respectful consideration of patients’ religious, ethnic, linguistic, and cultural identities fosters trust and confidence in the treatment team. Such culturally sensitive interaction is a key indicator of ethical competence in healthcare.

*“Patients have different religious attitudes and beliefs*,* and respecting their religious beliefs reduces stress and subsequently increases patients’ comfort and cooperation. Accordingly*,* it is essential to respect patients’ religious identity.” (Participant 18*,* 50 years old).*

Healthcare professionals also expressed that respecting patients with local dialects and accents is an essential ethical principle of competence.

*“There is a diversity of ethnicity and language in our country. Sometimes*,* some patients cannot speak the official language of the country well. To respect the ethnic and cultural identity of these patients*,* we request that staff who are fluent in the patient’s language speak to the patients and provide them with the necessary information.”) Participant 13*,* 27 years old.*

### Respecting physical and sexual privacy

The participants emphasized that upholding patient dignity by avoiding unnecessary exposure of the patient’s body and, whenever possible, assigning same-gender personnel reflects true ethical competence in the operating room.

“*A young woman entered the angioplasty room. I approached the patient and tried to prepare her for coronary angioplasty through the inguinal region. I brought the sterile Surgical Drapes after her permission and pulled them over her body*,* exposing only the right inguinal region that needed to obtain the arterial access. The patient appreciated my attention to her physical privacy and susceptible areas. Preserving sexual privacy is important for patients all over the world*,* and respecting the patient’s wishes and providing same-gender care conditions is appropriate*.”*) Participant 10*,* 44 years old.*

### Protecting information privacy

The study participants emphasized the importance of maintaining the confidentiality of patients’ medical records as one of the primary responsibilities of healthcare professionals in the operating room. Hence, the patients’ medical records must not be accessible to non-medical personnel and are only handed over to the nurse upon leaving the operating room.

*“Maintaining the confidentiality of patients’ information is the most crucial step in preserving their dignity. When healthcare professionals in the operating room strive to keep patients’ medical records only available to the treatment team and refrain from discussing patients with non-medical personnel*,* they have acquired one of the most critical factors in ethical professionalism.” (Participant 12*,* 47 years old)*.

### Theme 2: Promoting the patient’s mental health

The study participants emphasized that enhancing patients’ mental health in the operating room is a key indicator of healthcare professionals’ ethical competence. To improve patients’ mental well-being, it is essential to involve them in treatment decisions and provide comprehensive, understandable information before any procedure. Additionally, offering compassionate care helps alleviate anxiety and fear, enabling patients to feel secure and calm. These practices encompass three main aspects: respecting patients’ autonomy, providing thorough information, and demonstrating compassion.

### Paying attention to the patient’s independence

The participants emphasized the importance of involving patients in their treatment and care decision-making processes. When a situation is not an emergency, scheduling the procedure at a convenient time allows patients to prepare for it mentally. It is important to involve patients in decision-making regarding their medical interventions and surgical procedures. This not only helps to alleviate anxiety and increase mental health but also ensures that patients are fully informed about their options and have an idea of their healthcare.

*“Patients should be involved in the decision-making process for medical interventions and the timing of surgical procedures. The timing of the surgery should be determined based on the patient’s preferences. Patients involved in treatment decisions enter the operating room with less anxiety and greater mental health*.” (Participant 13, 27 years).

### Providing comprehensive information

Participants in this study expressed that providing comprehensive and transparent information to patients before any procedure can reduce their agitation, anxiety, and fear, and subsequently increase their cooperation. Therefore, avoiding rushed actions and providing understandable information in the cardiac operating room are essential for maintaining patients’ mental health.

“*The elderly man was anxious. I held his hand and explained the angioplasty procedure to him simply and understandably. He asked about the pain and immobility after the operation*,* and I explained* postoperative care to him and promised to explain more during the visits. Providing accurate and understandable information gives patients peace of mind and increases their trust.” (Participant 21, 36 years).

### Compassion

Healthcare professionals emphasize that compassion in the operating room—expressed through appropriate language and behavior—is fundamental to professional ethics and enhancing ethical competence. Greeting patients with a smile and treating them with kindness, especially in high-stress environments like cardiac surgery, helps alleviate anxiety and fosters a sense of security and calm. This compassionate approach is essential for upholding patients’ dignity and ensuring quality care.

*“I encountered a patient in the operating room who*,* despite being given explanations in the ward and having his questions answered*,* became anxious upon entering the operating room environment. I engaged in conversation with him and prepared him for the procedure. The patient said he felt more at ease*,* his fear subsided*,* and he felt safe. Showing empathy and compassion towards the patients in the operating room*,* especially in high-stress cardiac procedures*,* is fundamental for promoting patients’ mental health.” (Participant 9*,* 32 years old)*.

*“Patients undergoing angiography or angioplasty remain conscious during the procedure*,* and despite the administration of local anesthesia*,* some patients report pain. This phenomenon can potentially result in a distinct experience for patients and may lead to increased levels of stress and discomfort. “Patient anxiety during the procedure can increase sympathetic activity*,* leading to elevated heart rate and blood pressure. This physiological response reduces the quality of angiographic images*,* increases cardiac oxygen consumption*,* and elevates the likelihood of procedural complications*,* necessitating appropriate management through pharmacological interventions and adequate patient education. Effective communication with the patient*,* demonstrating empathy*,* and providing detailed explanations of the ongoing processes can contribute to the reduction of patient stress and anxiety.*” (Participant 5, 32 years).

### Theme 3: Expansion of moral obligations

According to the participants’ statements, ethical commitments play a crucial role in ensuring the quality of care and justice in healthcare. Key indicators of ethical commitment for healthcare professionals in the operating room include promoting ethical courage, avoiding discrimination, and fair policymaking. These themes can be further categorized as ethical courage, fair policymaking, and discrimination prevention.

### Promoting moral courage

Participants emphasized that promoting moral courage among healthcare professionals is essential for ethical competence. They advocated for fostering an environment where staff are encouraged to act courageously in supporting patients and defending their rights in the operating room. Cultivating this ethical courage is fundamental to developing ethical commitments, enhancing ethical competence, and fostering ethical dedication and proficiency.

“*Error is an inevitable part of medical treatment. Courage in reporting mistakes and shortcomings is a sign of commitment to ethical obligations in medical professions. I made a mistake regarding a patient’s illness. Although it was hard*,* I reported my mistake to my superior with all the details. I believe having courage is the most important indicator of ethical commitment and competence*” (Participant 6, 39 years old).

### Avoiding discrimination

Participants emphasized the importance of healthcare professionals refraining from discrimination between patients and treating them fairly and professionally. This represents ethical and human competence.

*“In government-operated healthcare facilities with insurance coverage*,* all patients should be afforded equal rights to access services. The patient’s economic*,* social*,* and political status should not influence the treatment process.” (Participant 3*,* 52 years old)*.

### Expanding ethical and principled care

Furthermore, it is imperative to acknowledge the patient’s right to undergo the procedure without experiencing any pain. It is essential to administer adequate anesthesia while recognizing that individual patients have varying requirements. The prescription of analgesics and anesthetics should be tailored to each patient’s specific needs because a standardized approach is not universally effective.

*“Patients with diverse physical conditions*,* including those with substance use disorders*,* require higher doses of analgesic and anesthetic agents compared to other patients. These dosages are prescribed based on the patients’ conditions before*,* during*,* and after the procedure.” (Participant 2*,* 42 years old)*.

### Promotion of ethical knowledge

Healthcare professionals indicated that continuous training of personnel, utilization of the latest scientific resources, and implementation of educational workshops were the primary factors contributing to the enhancement of their ethical knowledge.

*“Conducting educational workshops that utilize contemporary resources facilitates the acquisition of knowledge and the development of professional attitudes*,* subsequently enhancing the delivery of high-quality and safe care*,* which ultimately leads to increased patient satisfaction.” (Participant 10*,* 44 years old)*.

## Discussion

Research findings from healthcare professionals have demonstrated that the primary themes indicative of ethical competence in the cardiac operating room are “respect for the dignity of the patient,” “promoting the patient’s mental health,” and “expansion of moral obligations.”

One of the themes identified in this study was respect for patients’ dignity, which emphasized the importance of honoring their religious, ethnic, and cultural beliefs, maintaining their physical and sexual privacy, and preserving confidentiality. Consistent with the findings of the present study, other studies have highlighted the significance of respecting patients’ religious and cultural values as crucial elements in preserving their dignity within the healthcare team [10, 17]. According to Niknejad and colleagues (2019), respecting patients’ identities, perspectives, and spiritual and religious beliefs in the operating room creates a peaceful environment and preserves their dignity [10]. Mohammadi et al. (2020) emphasized the significance of respecting the religious and ethnic values and preferences of women undergoing surgery in the labor room as a crucial ethical principle for maintaining and enhancing patients’ dignity. This aspect is sometimes neglected, but is of great importance. Therefore, managers and policymakers must prioritize the implementation of ethical principles in the operating room, particularly for patients who are unconscious and rely solely on the treatment team for support [17]. A crucial aspect is respecting physical and sexual privacy during care. Park et al. (2025) emphasized the importance of respecting patients’ rights, including their sexual and physical privacy, as well as confidentiality [18]. Aghamohammadi et al. (2021) also emphasized that respect for patients’ privacy, especially their physical and sexual privacy, and providing care by same-sex individuals are among the most important ethical codes and principles that should be considered in the operating room [8]. Bakhtiari et al. (2020) highlighted the significance of upholding patients’ dignity and safeguarding their physical and sexual privacy as key ethical principles in the operating room [9]. Likewise, et al. (2022) recognized the importance of respecting and preserving the physical and sexual privacy of patients as a critical ethical competency for healthcare providers in the operating room [19]. Pattni et al. (2019) identified the invasion of patients’ physical and informational privacy without consent as a major ethical issue in operating rooms [20]. Respecting patient privacy is a crucial ethical principle in healthcare systems. Additionally, maintaining privacy in the operating room is another important theme, with various studies highlighting the importance of preserving patient privacy to uphold their dignity. In this regard, Mohammadi et al. (2020) state that preserving women’s privacy in the labor room and striving to keep their medical information confidential are professional principles [14]. Additionally, Pattni et al. (2019) also emphasized in their study that the most important ethical principles, along with preserving patients’ independence in the operating room, are efforts to keep their conversations with the treatment team confidential, which is consistent with the present study [20]. Öztürk et al. (2021) recognized that nurses respected patient privacy, but nurses working in training and research hospitals and internal disease clinics need to improve their approaches to patient privacy [21].

Another theme inferred from the present study was the improvement and promotion of patients’ mental health in the cardiac operating room. The participants emphasized that healthcare professionals should try to enhance patients’ mental health in the operating room. Additionally, they should strive to reduce fear and improve patients’ mental health through compassion and a warm reception. Consistent with the findings of Collopy et al. (2021), upskilling practitioners in mental health may be a further avenue to promote information provision and enhance service utilization [22]. According to Aghamohammadi et al. (2021), the ethical competence of healthcare professionals in the operating room involves shared decision-making, where patients participate in the diagnostic and therapeutic processes [8]. Furthermore, Bakhtiari et al. (2020) also highlighted the importance of considering the needs of patients in the operating room, especially their mental health needs, which is in line with the present study. Therefore, care teams need to pay attention to patients’ and their companions’ suggestions and priorities during the care process in the operating room [9]. These patients experience enhanced perceptions of value and efficacy when they are substantially involved in the process of diagnosis and treatment through shared decision making. This improves their psychological and emotional well-being, and subsequently, positive health outcomes [20]. Compassion and empathy towards patients are other important aspects of mental health promotion. Compassion and empathy are essential for reducing patient stress and establishing appropriate interactions. In line with the present study by Karo et al. (2023), nurses’ caring attitudes in the surgical room, which are based on caring, empathy, sensitivity, and responsibility in providing nursing care, can increase patient satisfaction, which can affect the quality of nursing services [23]. Babaei et al. (2021) also stated in their study that empathy activities and patient-centered emotional discourse are the main issues in shaping compassionate care in nurses [24]. Therefore, paying more attention to patients’ psychological needs and prioritizing compassion and empathy as ethical principles of greater professional competence are essential. Providing comprehensive information is another layer for promoting mental health in the cardiac operating room. It is appropriate to perform any intervention to increase mental health with the utmost calmness and after providing precise and comprehensive explanations, especially when the patients enter the cardiac operating room with high stress levels. Consistent with the findings of the present study, Mohammadi et al. (2020) stated that one of the most important principles of ethical competence in the operating room is providing detailed information before any intervention [14].

Another theme that draws on the ethical competence of healthcare in the cardiac operating room is the development of ethical commitment. In this regard, promoting ethical courage, avoiding discrimination, expanding ethical and principled care, and promoting ethical knowledge were the most critical factors in developing ethical commitments among healthcare professionals in the cardiac operating room. Consistent with the present study, Bakhtiari et al. (2020) also titled their study on the development of professional commitment, stating that personnel in the operating room should strive to promote ethical courage themselves to adequately support patients in the operating room who have no other support [9]. Additionally, Bruun et al. (2022) also stated that the student registered nurse anesthetist needs not only to learn about professional obligations and tasks but also to develop moral courage to respond to unethical behavior or communication in the operating room [14]. Avoiding discrimination is another vital class of inference drawn from this study, which destroys patients’ dignity and undermines their rights [14]. Consistent with this study, two other studies indicated that caregivers often discriminate among patients and overlook this ethical value [25, 26]. Therefore, avoiding discrimination is one of the most important ethical principles for healthcare professionals in operating rooms [9, 14, 25]. This study revealed that fair policies lead to the development of justice-oriented practices in healthcare. Teymoori et al. (2022) emphasized the importance of organizational culture and fair internal policies in advocating for patients’ rights and avoiding injustice, which aligns with the findings of this study [27]. Han et al. (2022) suggest that managers and policymakers should properly support patients’ and personnel’s rights by implementing precise and fair policies and determining benefits for personnel [28]. Chen et al. (2021) emphasize that acquiring ethical knowledge improves the level of ethical decisions [29]. Memarian et al. (2022) also stated that knowledge and skills, ethical conduct, professional commitment, self-respect, respect for others, and responsibility were effective in developing ethical behavior and subsequently nurses’ clinical competence [30].

### Limitation

One limitation of this study was its focus on healthcare professionals working in government-operated healthcare, which may restrict the generalizability of the findings. Furthermore, this study relied on individual interviews for data collection; incorporating additional qualitative methods, such as observation and focus groups, could provide more comprehensive insights into these experiences. Therefore, it is recommended that future studies examine healthcare professionals’ perceptions and experiences of ethical competence in the operating room, especially cardiac operating rooms, in private hospitals, and use a diverse range of qualitative data collection techniques.

## Conclusion

Healthcare professionals in the cardiac operating room define ethical competence as “respect for the dignity of the patient,” “promoting the patient’s mental health,” and “expansion of moral obligations.” Health managers and policymakers can make fundamental contributions to improving their ethical competence by utilizing the findings of this study and holding educational workshops in the areas of respecting patient dignity, improving patient mental health, and expanding ethical obligations for healthcare professionals in cardiac operating rooms.

## Data Availability

The datasets generated and/or analyzed during the current study are not publicly available due to the necessity of ensuring participant confidentiality policies and the country’s laws. Still, they are available from the corresponding author upon reasonable request.
